# The Impact of an Ice Slurry-Induced Gastrointestinal Heat Sink on Gastrointestinal and Rectal Temperatures Following Exercise

**DOI:** 10.3390/sports7090198

**Published:** 2019-08-27

**Authors:** Thomas A. Deshayes, Adrien De La Flore, Jonathan Gosselin, Jeff Beliveau, David Jeker, Eric D.B. Goulet

**Affiliations:** 1 Faculty of Physical Activity Sciences, University of Sherbrooke, Sherbrooke, QC J1K 2R1, Canada; 2 Research Centre on Aging, University of Sherbrooke, Sherbrooke, QC J1H 4C4, Canada; 3 Institut National des Sciences Appliquées, Université de Toulouse, 31400 Toulouse, France

**Keywords:** core body temperature, exercise, heat stress, ice slurry ingestion, telemetry, temperature measurement

## Abstract

Gastrointestinal temperature (*T*gint) measurement with a telemetric pill (TP) is increasingly used in exercise science. Contact of cool water with a TP invalidates *T*gint assessment. However, what effect a heat sink created in the proximity of a TP may have on the assessment of *T*gint remains unknown. We examined the impact of an ice slurry-induced heat sink on *T*gint and rectal temperature (*T*rec) following exercise. After 20 min of seating (20–22 °C, 25–40% relative humidity (RH)), 11 men completed two intersperse exercise periods (31–32 °C, 35% RH) at 75–80% of estimated maximal heart rate until a *T*rec increase of 1 °C above baseline level. Following the first exercise period, participants were seated for 45 min and ingested 7.5 g·kg^−1^ of thermoneutral water, whereas, following the second period, they ingested 7.5 g·kg^−1^ of ice slurry. Both *T*gint and *T*rec were measured continuously. The TPs were swallowed 10 h prior to the experiments. A bias ≤0.27 °C was taken as an indication that *T*gint and *T*rec provided similar core temperature indices. Mean biases and 95% limits of agreement during passive sitting, first exercise, water ingestion, second exercise, and ice slurry ingestion periods were 0.16 ± 0.53, 0.13 ± 0.41, 0.21 ± 0.70, 0.17 ± 0.50, and 0.18 ± 0.66 °C, respectively. The rates of decrease in *T*gint and *T*rec did not differ between the water and ice slurry ingestion periods. Our results indicate that ice slurry ingestion following exercise does not impact TP-derived assessment of *T*gint compared with *T*rec.

## 1. Introduction

The telemetric pill (TP) is being increasingly used by exercise scientists, physiologists, sports medicine physicians, and athletic trainers interested in determining and understanding the impact of various interventions on human thermoregulation during exercise [1]. The TP is typically swallowed several hours before data collection and therefore provides a measure of gastrointestinal temperature (*T*gint). The popularity of this measurement technique is not surprising, since there are obvious advantages associated with its use. For instance, compared to the more traditional, valid, and accepted method to assess core temperature during exercise, i.e., through the rectum via the use of a rectal probe [2], it is non-invasive, associated with little burden for users, can be used relatively easily under field conditions, and is not subject to negative preconceptions [3,4].

It has generally been accepted that *T*gint represents a reliable measurement of core temperature during exercise [3]. However, when the literature is closely scrutinized, one can observe that this is not always true, especially when cold fluids are being consumed. For example, Easton et al. [5] had participants ingest a TP 8 h prior to the commencement of the exercise trial and observed, on three occasions, *T*gint below 30 °C immediately following water (5 °C) ingestion. Similarly, Wilkinson et al. [6] observed that, 8 h following the ingestion of a TP, *T*gint could still decrease by as much as 6 °C immediately following water (5–8 °C) intake. During a half-marathon, Savoie et al. [4] had runners consume 200 mL of water (4 °C) every 2 km and observed that, as the run progressed, the disagreement between *T*rec and *T*gint slowly but steadily kept increasing, with a mean final disagreement between sites of 1.1 °C. This was observed even though TPs were ingested 10 h before experiments started. Albeit in the former two studies, measurement errors in *T*gint could be attributable to a direct contact of water with the TP, Savoie et al. [4] argued that, in their study, the creation of a heat exchanger surface may have been the culprit.

Gastrointestinal transit time may substantially vary within and between individuals [7]. Thus, following ingestion, the location of a TP within the gastrointestinal tract may be highly variable within and between individuals [8], and the closer a TP resides to a heat exchange medium at the upper gastrointestinal level, the higher the possibility that the temperature would be impacted. Ingestion of ice slurry has been demonstrated to decrease core temperature through the creation of a heat sink at the upper gastrointestinal level [9]. To this effect, Ihsan et al. [10] examined the effect of ice ingestion as a pre-cooling strategy on subsequent exercise performance. Participants ingested a TP 8–10 h prior to performance trials and consumed 6.8 g·kg^−1^ of either ice slurry or water (27 °C) 30 min before exercise. Water ingestion did not impact *T*gint, but during ice slurry intake, *T*gint decreased on average by 1.1 °C and, according to Lee [11], at least one individual showed a decrease in *T*gint below 35 °C. Similarly, Zimmerman et al. [12] had participants consume 7 g·kg^−1^ of either ice slurry or water (thermoneutral) over a 30 min period preceding exercise. Again, *T*gint was not impacted by water ingestion, but during the ice slurry intake, *T*gint decreased on average by 0.9 °C. Interestingly, studies have shown that the ingestion of 7.5 g·kg^−1^ of ice slurry does not decrease *T*rec by more than 0.6 °C [9,13,14,15]. Based on this observation, it is tempting to propose the idea that ice slurry ingestion may have more impact on *T*gint than *T*rec since the TP resides more closely to the heat sink than the rectal probe. However, we are aware of no studies that concurrently examine the kinetics of *T*gint and *T*rec following ice slurry ingestion.

Given the extent of the use of TP in the assessment of *T*gint, it is important to determine whether TP can reliably be used and provide good estimates of *T*gint under situations where heat sinks are created at the upper gastrointestinal level. Therefore, the goal of this study was to examine the impact of an ice slurry-induced heat sink following an exercise-induced increase in core body temperature on the degree of agreement between *T*gint and *T*rec. A greater impact of the heat sink on *T*gint than *T*rec would be reflected by a faster rate of decline in *T*gint than *T*rec and a lower absolute *T*gint than *T*rec following ingestion.

## 2. Materials and Methods

### 2.1. Participants

Eleven healthy and physically active men (32 ± 7 years; 1.77 ± 0.6 m; 74 ± 6 kg; body surface area: 1.91 ± 0.08 m^2^; resting heart rate: 57 ± 8 beats·min^−1^; and estimated maximal heart rate: 185 ± 5 beats·min^−1^) participated in this study. Participants were explained the study protocol and its associated risks, after which written informed consent was obtained. The University of Sherbrooke Institutional Review Board approved all protocol procedures (#2016-1144).

### 2.2. Overview of the Study

**Figure 1:**
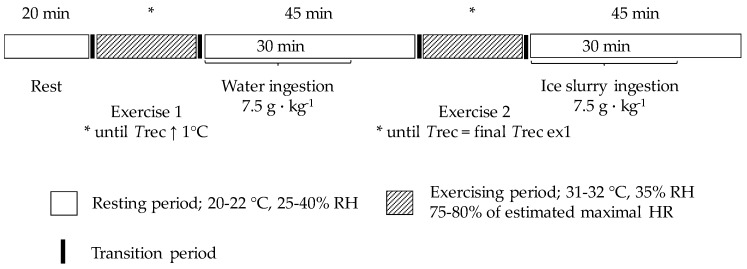
Schematic representation of the design of the study. HR: heart rate; *T*rec: rectal temperature; RH: relative humidity.

Participants came to the laboratory on two occasions (at 8:00 am for 9 participants and 04:30 pm for 2 participants). Baseline measurements were taken on the first visit, whereas during the second visit, participants underwent a research protocol where continuous measurements of *T*gint and *T*rec were obtained during a sequence comprising one sitting period, a running or a cycling exercise followed by water ingestion, and another running or cycling exercise followed by ice slurry ingestion (Figure 1).

### 2.3. Preliminary Visit

Participants’ height, body mass, resting heart rate, and blood pressure were measured during the preliminary visit. Post-void nude body mass and height were respectively measured using a digital scale (Bx-300+, Atron Systems, NJ, USA) and a wall stadiometer. After 3 min of seated rest, heart rate and blood pressure were taken with a digital sphygmomanometer (Welch Allyn 420 series, NY, USA). The equation developed by Tanaka et al. [16] was used to compute the estimated maximal heart rate. 

### 2.4. Pre-Experimental Protocol

For the experiment, all participants were asked to report to the laboratory in a well-fed and rested state. Also, they were requested to consume 250 mL of water 1 h before their arrival and then to remain fasted. The TP (CorTemp HQ Inc., FL, USA) was swallowed 10 h prior to arriving at the laboratory for all the participants. 

### 2.5. Experimental Protocol

Upon arrival at the laboratory, participants voided their bladder, provided a urine sample for urine specific gravity (USG) assessment, and their nude body mass was determined (Bx-300+, Atron Systems, NJ, USA). They then put on their running or cycling clothing and were instrumented with a chest electrode to measure heart rate, a rectal probe, and four epidermal probes affixed above the left pectoral muscle, the left forearm, the left thigh, and the left calf muscle. Then, the participants underwent the research protocol, which consisted of five phases that were performed sequentially: (1) a 20 min sitting period (20–22 °C, 25–40% relative humidity (RH)); (2) a running or a cycling exercise (75–80% of estimated maximal heart rate [16]) conducted at 31–32 °C with 35% RH aiming to increase *T*rec 1 °C above that measured at the end of the 20 min sitting period; (3) a 45 min sitting period while ingesting, over the first 30 min, 7.5 g·kg^−1^ of water (provided at mean *T*rec and *T*gint) every 10 min; (4) a second exercise period aiming to increase *T*rec 1 °C above that measured at the end of the 20 min sitting period; and (5) a 45 min sitting period while ingesting, over the first 30 min, 7.5 g·kg^−1^ of ice slurry (−1 °C) every 10 min. Participants could choose between a cycling or a running exercise, but the same mode of exercise as well as the same exercise intensity was used during the first and the second exercise bouts.

Transition times were not standardized between phases or participants but were performed as rapidly as possible. Participants could not consume fluids during the experiment other than the water and ice slurry provided during the sitting periods. However, they could rinse their mouths with cold water during the exercise periods. At the end of the experiment, participants voided their bladder, and a final nude body mass was taken.

### 2.6. Measurements and Procedures

Change in body mass from pre- to post-experiment period, corrected for water and ice slurry intakes and urine production, was used to estimate sweat loss. The dehydration level was estimated using the following formula:

Dehydration level: (∆ pre-post experiment body mass/pre-experiment body mass) × 100(1)

Urine specific gravity was measured using a digital refractometer (PAL-10S, Atago, WA, USA). Rectal and gastrointestinal temperatures were respectively measured with a calibrated YSI 401 wired rectal probe (Yellow Springs Instrument, OH, USA) and a TP (CorTemp HQ Inc., FL, USA). The wired rectal probe was inserted 15 cm beyond the anal sphincter and securely held in place with the aid of a lightweight harness. The YSI probe was connected to a high precision digital thermometer (Traceable 4005, Control Company, TX, USA). Gastrointestinal temperature signals were recorded with a single CorTemp Data Recorder (HQ Inc, Palmetto, FL, USA) held in place at the participants’ lower backs and base of the gluteal muscles. Both temperatures were recorded continuously every 20 s. The YSI probe and each TP were calibrated before the beginning of the experiment. Calibration was performed at four different temperatures (37, 38, 39, and 40 °C) in a heated bath (Precision 281, Thermo Scientific, MA, USA) using a high precision, partial immersion, non-mercury glass thermometer (Thermo Scientific Ertco, USA). A 4-point regression line [17] was used to predict *T*gint and *T*rec. Skin temperature was measured with YSI 409 B probes (Yellow Springs Instrument, OH, USA) and computed according to Ramanathan [18]. The four probes were held in place using Transpore tape (3 M, USA) and connected to a switch box linked to a high precision digital thermometer (Traceable 4005, Control Company, TX, USA). Throughout all five phases of the experiment, heart rate was measured with a T-31 Polar electrode linked to a Vantage NV Polar heart rate monitor (Polar USA, NY, USA). To produce the ice slurry, cubes of ice were shaved using a commercially available instrument (Hamilton Beach, USA). More precisely, the shaved ice was mixed with water (4 °C) in a 64:36% ice-to-water ratio. Moreover, ~22 g of glucose-fructose syrup (orange, grape, or raspberry) were added to the ice slurry in order to provide energy and increase palatability. Then, the ice slurry was conserved in the lower back of a refrigerator (2–4 °C). Finally, a few steps were necessary to produce the water provided at mean *T*rec and *T*gint. First, ~2.5 L of water were kept at 40 °C in a heated bath. Once the time came to produce the solution, ~5 g·kg^−1^ of this water were poured into an insulated bottle that contained a high precision digital thermometer. Then, rapidly but meticulously, 4 °C water was poured into the bottle until the desired temperature was obtained. Afterwards, the desired quantity of water, i.e., 2.5 g·kg^−1^, was transferred to another insulated bottle.

### 2.7. Statistical Analyses

Normality of data distribution was tested with the Shapiro–Wilk test. Acceptable agreement between measurement techniques was taken as a bias ≤0.27 °C [2]. Heart rate and mean skin temperature data were analysed using one-way repeated measures analysis of variance (ANOVA). Greenhouse–Geisser corrections were applied when sphericity was violated. Paired sample *t*-tests or Wilcoxon signed-rank tests were used to determine whether biases were systematic. A two-way repeated measures ANOVA was used to determine the effect of water and ice slurry conditions on *T*gint vs. *T*rec. Relative validity was assessed with the Pearson product-moment correlation coefficient (r). Absolute validity (random error statistics) was determined with the computation of the typical error of measurement (TEM), coefficient of variation (CV), and the Bland–Altman 95% limits of agreement (LoA). Heteroscedasticity was assessed using a linear regression model with *T*rec-*T*gint as the dependent variable and *T*rec-*T*gint/2 as the independent variable. Significant heteroscedasticity was observed for phases 3 to 5 and data comprising the entire experiment. However, no correction was performed given the weak relationship for each comparison. Given the different exercise times within and between participants, results for the exercise phases are reported based on percentage of maximal exercise time. Statistics were performed with the 2018 Microsoft Office Excel (Microsoft, WA, USA) and IBM SPSS Statistics (version 25, NY, USA) software. Results were considered significant at *p* ≤ 0.05. Data are reported as means ± SD unless stated otherwise.

## 3. Results

### 3.1. Temperature and Relative Humidity

The average temperature and the RH inside the heat chamber were respectively 31.7 ± 1.3 °C and 33.1 ± 5.5% for the first exercise period and 30.8 ± 1.1 °C (*p* = 0.10) and 33.5 ± 5.3% (*p* = 0.71) for the second exercise period. Sitting periods were performed at an ambient temperature of 20–22 °C with 25–40% RH.

### 3.2. Fluid Balance

Participants were well hydrated before the start of the experiment, as supported by a mean USG of 1.016 ± 0.009 g·mL^−1^. Mean USG increased to 1.021 ± 0.005 g·mL^−1^ at the end of the experiment (*p* = 0.01). Average ice slurry and water intake was 555 ± 41 g. Following exercise, urine production, sweat loss, sweat rate, and dehydration level amounted to 256 ± 122 mL, 1593 ± 441 mL, 540 ± 96 mL·h^−1^ and 1.0 ± 0.7% of body mass, respectively.

### 3.3. Exercise Time, Heart Rate, and Exercise Intensity

**Figure 2:**
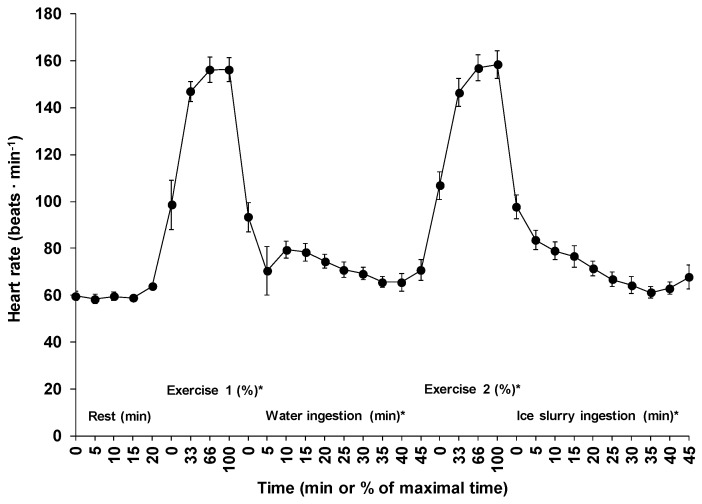
Changes in heart rate across time for all five phases of the experiment. Results are means ± SD. *: significant time effect throughout the phase.

The mean exercise times for the first and the second exercise periods were 24.8 ± 9.5 and 25.0 ± 13.6 min (*p* = 0.72), respectively. Figure 2 shows the changes in heart rate across time for all five phases. Average heart rate during the first (152 ± 12 beats·min^−1^) and the second (153 ± 15 beats·min^−1^) exercise periods corresponded respectively to 78 ± 5 and 78 ± 6% of estimated maximal heart rate (*p* = 0.53). Except for the 20 min passive sitting period (*p* = 0.18), a significant time effect was observed for the changes in mean heart rate across time for all other phases (*p* < 0.01).

### 3.4. Mean Skin Temperature

**Figure 3:**
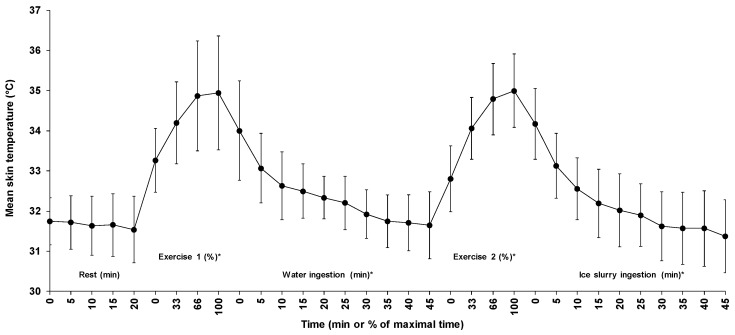
Changes in mean skin temperature for all five phases of the experiment. Results are means ± SD. *: significant time effect throughout the phase.

Figure 3 illustrates the changes in mean skin temperature across time for all five phases. Average skin temperature during passive sitting, first exercise, water ingestion, second exercise, and ice slurry ingestion periods were 31.7 ± 0.7, 34.3 ± 1.2, 32.4 ± 0.8, 34.2 ± 0.9, and 32.2 ± 0.9 °C, respectively. Except for the 20 min passive sitting period (*p* = 0.27), a significant time effect was observed for the changes in mean skin temperature across time for all other phases. The average mean skin temperature increased by 1.7 ± 1.1 and 2.2 ± 0.9 °C during the first and the second exercise periods, respectively (*p* < 0.01) and decreased by 2.3 ± 1.6 and 2.8 ± 1.2 °C, respectively, during the water and the ice slurry ingestion periods (*p* < 0.01).

### 3.5. Heat Sink

**Figure 4:**
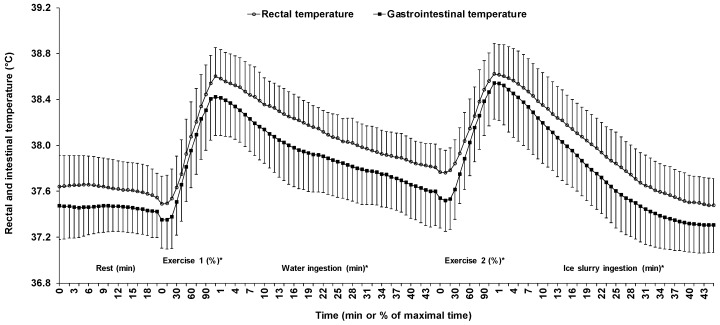
Changes in gastrointestinal and rectal temperatures across time for all five phases of the experiment. Results are means ± SD. *: significant time effect throughout the phase.

The heat sink was successfully created, as evidenced by a steeper decrease in both *T*gint and *T*rec during the ice slurry than the water ingestion period (Figure 4). On average, during the water ingestion period, *T*gint decreased by 0.82 ± 0.35, whereas *T*rec decreased by 0.79 ± 0.16 °C. On the other hand, during the ice slurry ingestion period, *T*gint decreased by 1.24 ± 0.27, whereas *T*rec decreased by 1.15 ± 0.24 °C. Altogether, ice slurry ingestion further decreased *T*gint by 0.41 ± 0.40 compared with 0.35 ± 0.21 °C for *T*rec (*p* = 0.72). The rates of decrease of both *T*gint and *T*rec were faster (*p* < 0.01) in the ice slurry than the water condition.

### 3.6. Gastrointestinal and Rectal Temperatures

Figure 4 presents the changes in *T*gint and *T*rec across time during the five phases. Mean *T*gint and *T*rec during the entire experiment were respectively of the order of 38.0 ± 0.4 and 37.8 ± 0.5 °C (*p* < 0.01). With the exception of *T*gint during the passive sitting period, a significant time effect was observed across all five phases with, as expected, a decline in mean *T*gint and *T*rec during the water and the ice slurry ingestion periods, with the opposite occurring during both exercise periods (all *p* < 0.01). The rates of change in *T*gint and *T*rec across all five phases were similar with values of −0.002 ± 0.008 vs. −0.005 ± 0.006 (*p* = 0.50), 0.047 ± 0.020 vs. 0.047 ± 0.017 (*p* =0.92), −0.018 ± 0.008 vs. −0.018 ± 0.004 (*p* = 0.17), 0.041 ± 0.022 vs. 0.036 ± 0.012 (*p* = 0.74), and −0.028 ± 0.006 vs. −0.026 ± 0.005 °C·min^−1^ (*p* = 0.47) for phases 1, 2, 3, 4, and 5, respectively.

Data related to the relative and the absolute comparisons between *T*gint and *T*rec are reported in Table 1. For all five phases, *T*rec was greater than *T*gint (all *p* < 0.01), with all biases being systematic, including the global bias (*p* < 0.01). In none of the five phases was the mean bias between *T*rec and *T*gint greater than the a priori established threshold of ≤0.27 °C. There was a pattern where the agreement between *T*rec and *T*gint was stronger during the exercise than the resting periods. In fact, biases, CVs, TEMs, and 95% LoAs were lower during the former than the latter periods, with little variation between the water and the ice slurry ingestion periods. Pearson product-moment correlation coefficients followed the same pattern and were higher during the exercise than the resting periods. However, the relationship between *T*rec and *T*gint was stronger with ice slurry than water ingestion.

## 4. Discussion

The aim of this study was to determine whether TP-derived *T*gint and rectal probe-derived *T*rec are impacted differently by the creation of an ice slurry-induced heat sink following exercise.

Participants were requested to complete two bouts of exercise, after which, on one occasion, thermoneutral water was consumed and, on the other, ice slurry was consumed. The goal of the water trial was to characterize the pattern of decline in *T*gint and *T*rec while considering the effect of fluid ingestion on thermal and non-thermal effectors of body heat loss [19]. Gastrointestinal and rectal temperature responses were examined after rather than during exercise to reduce the potential confounding effect of (1) gastric emptying on the rate of fluid transfer to the intestines [20]; (2) thermal inertia, which may have resulted in different amounts of body heat storage among conditions [21]; and (3) movement-induced gastric motility. The differences between *T*rec and *T*gint during the thermoneutral water and the ice slurry ingestion periods were similar and ≤0.27 °C, our a priori established threshold for non-difference between sites. From a practical standpoint, these results indicate that, following exercise, the monitoring of *T*gint is unlikely to be impacted by a cooling of the upper gastrointestinal tract induced by a mass of ice slurry of about 555 g·74 kg body mass^−1^.

Rectal temperature was systematically higher than *T*gint throughout the different phases of the experiment. Previously published studies have reported conflicting findings regarding the direction of the bias between *T*rec and *T*gint during multi-phase experiments comprising resting and exercising periods with or without fluid ingestion [1]. For instance, Casa et al. [2] (0.19 °C), Kolka et al. [22] (0.18 °C), and Gosselin et al. [23] (0.20 °C) reported higher *T*rec than *T*gint. On the other hand, Gant et al. [24] (0.15 °C) and Teunissen et al. [25] (0.14 °C) observed *T*gint to be higher than *T*rec. Our findings add to the current literature and indicate that an acceptable difference between *T*rec and *T*gint during experiments composed of various passive and active phases with or without fluid or ice slurry ingestion should lie within ±0.20 °C, which is reasonable from a technical and a physiological point of view given that the daily variability in *T*rec is ~±0.25 °C [26].

Although the overall mean bias (0.18 °C) between *T*rec and *T*gint was under what we consider to be an acceptable difference between sites, i.e., <0.27 °C, the mean 95% LoA (±0.63° C) indicates that there may be wide variations between sites within and between individuals. Given that the bias was in favor of a greater *T*rec than *T*gint over the entire research protocol, as illustrated in Figure 4, this may have an important implication for the practitioners or the coaches using TPs to monitor core temperature. Indeed, in the case of a suspected exertional heat stroke, our findings indicate that *T*rec should also be taken instead of relying solely on *T*gint in order to establish the correct diagnostic and initiate the proper treatment.

Whether in absolute or relative terms, the patterns of temporal changes in temperature between *T*rec than *T*gint during the water and the ice slurry ingestion periods were similar, thereby implying that ice slurry ingestion did not impact *T*gint differently than *T*rec. Several reasons may be thought of to explain this observation. The amount of cooling in the face of hyperthermia may not have been important enough such that thermal equilibrium with the surrounding tissues was attained prior to reaching the TPs. The volume of warm blood returning to the upper gastrointestinal area, and hence convective heat flow, may have been more important than in the rectal area, therefore hiding any potential effect of the heat sink on *T*gint. Moreover, a higher metabolic rate (driven by fluid absorption) of the upper intestinal region than in the rectum would have acted to increase local temperature and therefore mask the effect of the heat sink. However, the two preceding scenarios are improbable, as this would have been reflected during the water ingestion period as a lower rate of decline in *T*gint than *T*rec, which was not the case. A greater thermal inertia of the TP is unlikely to have been responsible for the observed findings. In fact, Gosselin et al. [23] demonstrated that, when inserted concurrently in the rectum, the absolute and the relative changes in temperature between a TP and wired rectal probe are similar.

An interesting observation of this study is that the divergence in temperatures between *T*rec and *T*gint was more important during the ingestion periods than during the exercise periods, as underlined by higher TEMs, CVs, and 95% LoAs during the former than the latter period. During exercise, the sympathetic activity-driven arteriole vasoconstriction was likely similar between the rectal and the upper gastrointestinal area, thereby producing a more uniform response between regions and among individuals due to a greater reliance on conduction than convection for heat exchange. On the other hand, following exercise, heat exchange with surrounding tissues became more reliant upon convection, and the rate as well as the total amount of blood perfusing the rectal and the upper gastrointestinal area likely differed among individuals, explaining the greater variability in results.

The present findings need to be interpreted with the following limitations in mind. First, we provided a mass of ice slurry of 7.5 g·kg^−1^, which created an estimated heat debt of 210 kJ. It is not impossible that a higher mass of ice slurry would have unraveled a difference between *T*rec and *T*gint. However, from a practical perspective, consumption of a higher mass of ice slurry would have been difficult to sustain by participants within the 30 min timeframe allowed for ingestion. Moreover, the amount of ice slurry administered is in line with the mass most studies examining the impact of ice slurry ingestion on thermoregulation have provided, which enhances the external validity of our findings. Second, the exact location of the TP within the gastrointestinal tract was unknown at the time of the water and the ice slurry ingestion periods. Since the TPs were ingested 10 h prior to starting the experiments, it is therefore reasonable from a physiological standpoint to believe that they may have been at a distance too far from the heat exchange surface to be impacted differently than *T*rec. However, timing of ingestion of the TPs was deliberately chosen to optimize the external and the ecological validity of findings, as it has been suggested that it is the optimal timeframe for preventing external contamination from fluid or food consumption [4]. Finally, the created heat sink could exchange energy with all tissues surrounding its center. However, as the TPs were located beneath the center of the heat sink, only a certain proportion of tissues impacted by the heat sink could exchange heat with the sensors.

In conclusion, the present results indicate that the ingestion of ice slurry following exercise does not impact measurement of *T*gint compared with *T*rec, given that the ingestion of the TP occurs 10 h prior. These results have implications for athletic trainers, sports medicine physicians, scientists, physiologists, and military personnel who use TPs to monitor core temperature changes following exercise using a variety of cooling procedures, including ice slurry ingestion at a mass of 7.5 g·kg body mass^−1^.

## Figures and Tables

**Table 1:** Comparisons between gastrointestinal and rectal temperatures.

Periods	Bias (°C) [95% CI]	TEM (°C)	CV (%)	95% LoA (°C)	*r*
Passive sitting	0.16 [0.13–0.20] *	±0.27	±0.40	±0.53	0.33
Exercise 1	0.13 [0.09–0.16] *	±0.21	±0.33	±0.41	0.91
Water ingestion	0.21 [0.18–0.24] *	±0.36	±0.58	±0.70	0.48
Exercise 2	0.17 [0.12–0.21] *	±0.25	±0.40	±0.50	0.81
Ice slurry ingestion	0.18 [0.15–0.21] *	±0.34	±0.60	±0.66	0.77
Mean	0.18 [0.17–0.20] *	±0.32	±0.52	±0.63	0.73

Bias: rectal-intestinal temperature; CI: confidence interval; CV: coefficient of variation; LoA: 95% limits of agreement; *r*: Pearson product-moment correlation coefficient; TEM: typical error of measurement; *: systematic bias.
